# Dose‐dependent short‐ and long‐term effects of ionizing irradiation on neural stem cells in murine hippocampal tissue cultures: neuroprotective potential of resveratrol

**DOI:** 10.1002/brb3.548

**Published:** 2016-08-12

**Authors:** Isabell Prager, Ina Patties, Katrin Himmelbach, Eva Kendzia, Felicitas Merz, Klaus Müller, Rolf‐Dieter Kortmann, Annegret Glasow

**Affiliations:** ^1^ Department of Radiation TherapyUniversity of LeipzigLeipzigGermany; ^2^ Institute of AnatomyUniversity of LeipzigLeipzigGermany

**Keywords:** hippocampus, ionizing radiation, nestin, organotypic slices, resveratrol

## Abstract

**Introduction:**

Radiation therapy plays an essential role in the treatment of brain tumors, but neurocognitive deficits remain a significant risk, especially in pediatric patients. In recent trials, hippocampal sparing techniques are applied to reduce these adverse effects. Here, we investigate dose‐dependent effects of ionizing radiation (IR) on juvenile hippocampal neurogenesis. Additionally, we evaluate the radioprotective potential of resveratrol, a plant polyphenol recognized for its bifunctional tumor‐preventive and anticancer effects.

**Methods:**

Organotypic entorhinal–hippocampal slice cultures from transgenic nestin‐CFPnuc C57BL/J6 mice, postnatal days 3–6, were irradiated on a X‐ray machine (4.5, 8, 12, and 16 Gy, single doses) after about 2 weeks. Nestin‐positive neural stem cells were counted at a confocal live imaging microscope 0, 2, 4, 14, 25, and 42 days after IR. Resveratrol (15 μmol/L) was added 2 hr before and 24 hr after IR. Proliferation and cell death were assessed by BrdU pulse label, 48 hr after and by propidium iodide staining 96 hr after IR. GFAP‐ and NeuN‐positive cells were counted 42 days after IR in cryosectioned immunofluorescence‐stained slices.

**Results:**

The observed age‐related changes of nestin‐positive stem cells in the organotypic slice culture model resembled the reduction of neural stem cells in vivo. IR (4.5–16 Gy) led to a dose‐dependent damage of the neural stem cell pool in the dentate gyrus. No recovery was seen within 42 days after doses from 4.5 Gy onward. The decline of nestin‐positive cells was paralleled by increased cell death and decreased proliferation. The number of GFAP‐positive cells was significantly enhanced. No significant change was detected in the overall NeuN‐positive cell population, whereas the number of newborn, NeuN/BrdU double‐positive neurons was reduced. Resveratrol treatment reversed the irradiation‐induced decline of neural stem cells.

**Conclusion:**

The neuroprotective action of resveratrol on irradiated hippocampal tissue warrants further investigation as a possible supplement to hippocampal sparing procedures.

## 1 Introduction

Brain tumors are the second most common childhood malignancies. Excellent survival rates have been achieved in some entities using multimodular treatment approaches which contain radiation therapy as integral component. However, radiation‐induced adverse reactions to the normal tissue remain a major problem. Irradiation is currently considered the most important risk factor for the deterioration of neurocognitive functions (Duffner, 2010; Redmond et al., 2013). A meta‐analysis assessing effects of 18, 24, and 36 Gy whole and partial brain radiation therapy came to the conclusion that lower doses resulted in improved intelligence quotient, especially in children <6 years (Fuss, Poljanc, & Hug, 2000). Also studies in medulloblastoma patients aged 6–14 years recorded a negative impact on the intellectual outcome (Moxon‐Emre et al., 2014; Palmer et al., 2013). A prospective trial showed that the extent of radiation‐induced cognitive deficits in pediatric low‐grade glioma patients was age‐dependent and also cumulative over time after application of 54 Gy. In children <5 years, the greatest cognitive decline was observed (Merchant, Conklin, Wu, Lustig, & Xiong, 2009). As a consequence, the German guidelines of pediatric oncology and hematology recommend to avoid radiation therapy in very young (under 3–5 years of age) medulloblastoma patients (http://www.awmf.org/uploads/tx_szleitlinien/025-009l_S1_Medulloblastom_2012-11.pdf).

The hippocampus is the anatomical substrate for major neurocognitive abilities (declarative memory, spatial orientation, and learning). Accordingly, damage to the hippocampus plays a central role in the pathogenesis of radiation‐induced neurocognitive deficits (Abayomi, 1996). The subgranular zone (SGZ) of the hippocampal dentate gyrus is the main site of postnatal neurogenesis beside the subventricular zone (SVZ) (Eriksson et al., 1998). In both brain stem cell niches, new neurons arise throughout life (Bergmann, Spalding, & Frisen, 2015; Kempermann, Song, & Gage, 2015; Lepousez, Nissant, & Lledo, 2015), possibly providing a pool for tissue regeneration after brain injury. Cranial radiation experiments in rats showed a better long‐term recovery of neural stem cells in the SVZ than in dentate gyrus of the hippocampus (Hellstrom, Bjork‐Eriksson, Blomgren, & Kuhn, 2009), underlining the necessity of radioprotection in the hippocampus. Monje et al. (2007) could show at human hippocampi that neurogenesis is ablated after radiation treatment for acute myelogenous leukemia or medulloblastoma. Recent experimental studies on neurogenesis mostly in adult mice, demonstrated a dose‐dependent decline of doublecortin‐positive proliferating neural progenitor cells after whole body irradiation (0.5–4 Gy), which correlated with significant learning and memory shortcomings in specific behavioral tests (Kim et al., 2008) and reviewed in Andres‐Mach, Rola, and Fike (2008).

A promising approach to improve neurocognitive outcome after irradiation is therefore to minimize the dose burden of the hippocampus (“hippocampal sparing”) (Oskan et al., 2014). A feasibility study of Gondi et al. (2010) assessing hippocampal sparing techniques in cranial radiation therapy showed that the median dose can be lowered from 30 Gy to less than 8 Gy. The risk of peri‐hippocampal metastases remained within acceptable limits. In a phase II trial for adults with brain metastases, an association of hippocampal sparing with memory preservation was found (Gondi et al., 2014).

As the technical potential to reduce the radiation burden to certain organs at risk is limited, neuroprotective agents may be a useful supplement to hippocampal sparing to further minimize the detrimental effects of irradiation on hippocampal neurogenesis and neurocognition (Rooney & Laack, 2013). The natural polyphenol resveratrol has attracted our interest because of its anticancer and cancer‐preventive properties. Resveratrol was found to act as an antioxidant and antimutagen, promotes cell differentiation in leukemia cells (Jang et al., 1997), and is able to induce cell cycle arrest and apoptosis in human medulloblastoma cells (Zhang et al., 2006). Interestingly, also radiosensitizing effects of resveratrol have been shown in medulloblastoma (Patties, Jahns, Hildebrandt, Kortmann, & Glasow, 2009) and Merkel cell carcinoma cell lines (Heiduschka et al., 2014).

Resveratrol has also the ability to protect neuronal cells through regulation of inflammatory and apoptotic events (Mohamed, El‐Swefy, Hasan, & Hasan, 2014). Moreover, resveratrol has been shown to inhibit radiation‐induced apoptosis in the hippocampus (Li et al., 2014), and to antagonize the effects of reactive oxygen species in neuronal cells (Fukui, Choi, & Zhu, 2010).

In this study, we investigated (1) the short‐ and long‐term dose–response relationship underlying radiation‐induced impairment of juvenile hippocampal neurogenesis and (2) the neuroprotective potential of resveratrol. The applied dose range (4.5–16 Gy, single dose) was chosen to cover “hippocampal sparing” as well as conventional doses. We hypothesized that neuronal precursor cells would be damaged irreversibly by higher doses of irradiation and that this damage could be modified by the application of resveratrol.

We used organotypic entorhinal–hippocampal slice cultures (OEHSCs) (Gahwiler, Capogna, Debanne, McKinney, & Thompson, 1997; Kluge, Hailer, Horvath, Bechmann, & Nitsch, 1998) of transgenic nestin‐CFPnuc C57BL/J6 mice, generated by Encinas, Vaahtokari, and Enikolopov (2006) as an ex vivo system for the study of neurogenesis. Nestin is an intermediate filament protein typically expressed in neural progenitor cells (Encinas et al., 2006; Lendahl, Zimmerman, & McKay, 1990). The nuclear localization of nestin in the aforementioned mouse line strongly facilitates accurate enumeration of neural progenitor cells (Encinas et al., 2006). The biological environment in pediatric patients was reproduced by the age of the animals (postnatal days [p] 3–6) (Flurkey & Currer, 2004). The slice model retains tissue functions and cytoarchitecture as present in living animals (Gahwiler, 1984). The perforant path remains intact (Kluge et al., 1998), enabling the communication between the dentate gyrus and the entorhinal cortex, which is a prerequisite for the long‐term survival of the hippocampal slices. Furthermore, the model allows the quantification of nestin‐positive neural stem cells over long time periods by live imaging microscopy as well as easy drug application and irradiation. The number of experimental animals could be significantly reduced as up to eight hippocampal slices were obtained from a single animal.

## 2 Materials and Methods

### 2.1 Animals

Nestin‐CFPnuc C57BL/J6 mice (Encinas et al., 2006) originally from G. Enikolopov (Cold Spring Harbor Laboratory, CSH, NY 11724, US) were kindly provided by G. Kempermann, (CRTD‐DFG, Technische Universität Dresden, DZNE, Dresden, Germany). Mouse breeding was performed in the animal facility of the Faculty of Medicine, University of Leipzig according to European (Council Directive 86/609/EEC) and German (Tierschutzgesetz) guidelines for the welfare of experimental animals. Nestin‐CFPnuc C57BL/J6 mice were housed in a 12‐hr/12‐hr light/dark cycle with access to food and water ad libitum. All experiments had been approved in advance by the local authorities (Landesdirektion Sachsen T100/13).

### 2.2 Preparation of murine entorhinal–hippocampal slice cultures

Organotypic entorhinal–hippocampal slice cultures (OEHSC) were generated from nestin‐CFPnuc C57BL/J6 mice, postnatal day (p) 3 to p6. For that, a method, initially described by Gahwiler et al. (1997) and modified by Kluge et al. (1998), was established. Briefly, after decapitation of the mice, brains were placed in ice‐cold preparation medium consisting of MEM (Invitrogen) with 1% l‐glutamine (Sigma), 1% penicillin/streptomycin (Lonza), and 1% glucose (stock solution 45%, Applichem), and the frontal pole was removed. Beginning with the ventral surface, 350‐μm thick horizontal slices were cut on a vibratome (Leica VT 1000) under sterile conditions. Up to four slices (eight hippocampi) per mouse were collected. The entorhinal–hippocampal formation which contains the dentate gyrus was resected from the surrounding tissue under light‐microscopic control and transferred onto porous membrane inserts (Millicell PICMORG50, Millipore) in six‐well culture plates. The cultivation medium consisted of MEM (Invitrogen) with 25% Hank's balanced salt solution (Invitrogen), 25% horse serum (Invitrogen), 1% l‐glutamine (Sigma), 1% penicillin/streptomycin (Lonza), and 1% glucose (stock solution 45%, Applichem). Slices were cultivated at 37°C and 5% CO_2_. Medium was changed three times per week. Serum‐free medium (neurobasal‐A medium with B27 complement, 5 mmol/L glucose, and 2.5 mmol/L l‐glutamine) was only applied for a control experiment to analyze the serum effect on nestin expression.

### 2.3 Application of BrdU

To analyze the cell proliferation in response to irradiation, slices were pulse‐labeled with 0.2 μmol/L 5‐bromo‐2‐deoxyuridine (BrdU, Roche) which is incorporated into the DNA during the s‐phase (length 8.0 ± 0.4 hr) of the cell cycle. BrdU was added to the culture medium 2 hr before irradiation and removed 48 hr later by medium change. The estimated length of the cell cycle of the cells comprising the intrahilar proliferative zone is 16.1 ± 0.8 hr (Kalm, Karlsson, Nilsson, & Blomgren, 2013) so that the cells could divide approximately two times after exposure to irradiation until BrdU was removed. Proliferation rates were determined by immunofluorescence staining 14 days after irradiation.

### 2.4 Application of resveratrol

Two hours before irradiation (0, 8 or 16 Gy), resveratrol (15 μmol/L, Sigma) was added to the culture medium and renewed the next day by changing the culture medium and adding resveratrol again. Forty‐eight hours after irradiation, the culture medium was removed and substituted by normal culture medium. Resveratrol was dissolved in ethanol (0.01%). Control slices were treated with ethanol at the same concentration.

### 2.5 Irradiation

Irradiation was conducted 2 weeks after slice generation (=d 0). After this wound‐healing period, cell culture plates were wrapped in parafilm for irradiation. Slices were irradiated with a single shot of 4.5, 8, 12, or 16 Gy using a 150‐kV X‐ray unit (DARPAC 150‐MC) with a dose rate of 0.86 Gy/min. Sham‐irradiated control slices (0 Gy) were treated at equal conditions as irradiated slices.

### 2.6 Live imaging analysis of neural progenitor cells and cell death

Nestin, a common marker of the neural progenitor population (Lendahl et al., 1990), was visualized by its CFPnuc label with an Olympus BX51 confocal fluorescence microscope at 458 nm excitation. After placing the tissue cultures in a chamber (60% humidity, 37°C, 5% CO_2_), the dentate gyrus could easily be detected in each slice due to the fluorescence signal of nestin‐CFPnuc‐positive cells. The structure was centered to the visual field and imaged at 20‐fold magnification before irradiation (d 0, control) as well as 2, 4, 14, 25/28, and 42 days after irradiation. Z‐stacks of three images at intervals of 4 μm were obtained. For quantification of fluorescent cells, ImageJ and the Plugin Cell Counter (http://imagej.nih.gov/ij/) were used. The parameters for imaging were kept constant for each dataset.

Cell death within the dentate gyrus was assessed by propidium iodide (PI) uptake 96 hr after irradiation. PI (Sigma) was added to the culture medium to a final concentration of 5 μg/mL 2 hr before live imaging started and removed afterward by medium change. PI‐positive cells were visualized using the 543 nm laser line of the confocal microscope and quantified as described above.

### 2.7 Immunofluorescence staining and quantification of neuroglia, mature neurons, and proliferation

For all immunofluorescence stainings, inserts were rinsed in phosphate‐buffered saline (PBS, Lonza) 14/42 days after irradiation and fixed with 4% paraformaldehyde (Roth) in PBS at 4°C for 4 hr. Slices were removed from the insert, washed in PBS, and transferred into sucrose (Applichem, 15% in PBS) at 4°C overnight and for further 4 hr in 30% sucrose at room temperature. Slices were then covered with Tissue‐Tek OCT compound on a frozen stage in a cryostat (Leica CM 1520), cut at −26°C into 8μm horizontal sections and stored at −80°C. Stainings were performed at room temperature if not otherwise mentioned.

For NeuN staining, sections were washed in PBS, permeabilized with 0.5% Triton X‐100 at 4°C for 5 min, and immersed in PBS with 10% normal goat serum for 30 min to block unspecific binding. Sections were then incubated with the primary antibody (mouse anti‐NeuN, Millipore Cat# MAB377, RRID:AB 2298767) 1:100 in PBS with 2% normal goat serum for 1 hr, and after three washes in PBS, with the secondary antibody (Alexa 680‐conjugated goat anti‐mouse IgG F(ab’)2, Invitrogen) 1:500 in PBS with 2% normal goat serum for another hour.

GFAP and Ki‐67 (an antigen expressed in all phases of the cell cycle except G_0_) staining was performed according to NeuN staining except for following specifications: for GFAP, blocking solution contained 10% normal goat serum in PBS and 0.2% Triton X‐100. Antibodies used for GFAP were primary antibody (mouse anti‐GFAP IgG2b, 1:1000, BD Biosciences Cat# 556330, RRID:AB_396368) and secondary antibody (Alexa 680‐conjugated goat anti‐mouse IgG F(ab’)2, 1:500, Invitrogen). For Ki‐67, slices were cooked for 5 min in antigen retrieval buffer (10 mmol/L Tris/1 mmol/L EDTA, 0.05% Tween 20, Roth), left to cool down for 20 min, washed twice in PBS, and incubated for 30 min in blocking solution (PBS with 2% normal goat serum). Antibodies for Ki‐67 were primary antibody (mouse anti‐human Ki‐67 IgG_1_, 1:50, BD Biosciences Cat# 556003, RRID:AB_396287) and secondary antibody (Alexa 680‐goat anti‐mouse IgG F(ab’)2, 1:200, Invitrogen). Nuclei were counterstained with DAPI (4′6‐diamidino‐2‐phenylindole‐dilactate 10 mg, 1:10.000, Invitrogen) for 5 min.

For double staining with BrdU, first staining (NeuN) was fixed by incubation in paraformaldehyde, 4% in PBS, for 15 min at 4°C, followed by two washes with 0.9% sodium chloride. BrdU staining started by incubation with hydrochloric acid, 2 N, at 37°C for 30 min to denature the DNA. After rinsing in PBS and blocking (10% normal goat serum in PBS with 0.2% Triton X‐100) for 30 min, they were incubated with the primary antibody (rat anti‐BrdU, IgG2a, 1:1000, AbD Serotec Cat# MCA2060T, RRID:AB_10015293) in PBS with 2% normal goat serum and 0.2% Triton X‐100 at 4°C overnight. After three washes with PBS, sections were incubated with the secondary antibody (Alexa 568‐conjugated goat anti‐rat IgG, 1:1500, Invitrogen) in PBS with 2% normal goat serum for 1 hr.

For all stainings, specific IgG isotype controls (NeuN, Ki67: mouse monoclonal IgG_1_, 1:50, BD Pharmingen; BrdU: ratIgG2a, 1:500, eBioscience; GFAP: mouse IgG2b, 1:200, Abcam) were applied.

Sections were mounted in Mowiol 4‐88/DABCO (Roth, Sigma) and ≥3 sections per slice analyzed at 20‐fold or 40‐fold magnification on a fluorescence microscope (ZEISS AXIO Lab drb KT 450905 and AxioCam MRm) using ZEN 2012 blue edition software. Cell counting in the dentate gyrus region includes the SGZ. The results were expressed as the number of immunolabeled cells per dentate gyrus if not otherwise noted.

### 2.8 Hematoxylin–eosin staining

Paraformaldehyde or ethanol/acetone (1:1)‐fixed sections were stained with hematoxylin 1% (Sigma, MHS 16) for 25 min and blued for 15 min with tap water. After washing in 99% ethanol for 40 s, sections were incubated in eosin 0.1% (Sigma) for 1 min, rinsed with 99% ethanol (twice), and mounted in Entellan (Merck).

### 2.9 Cytokine measurements

Supernatants of slice cultures were analyzed for markers of inflammation. A vacuum centrifuge (SPD111V speedvac concentrator, Savant) was used to concentrate 100 μL samples fourfold. Interleukin‐6 (IL6), keratinocyte chemoattractant (KC, CXCL1), and macrophage chemoattractant protein‐1 (MCP‐1) were then measured by cytometric bead array (CBA, BD Biosciences).

### 2.10 Statistical analysis

All (sham) treatments were performed on randomly selected slices of the same mouse and repeated in *n* mice allowing a paired statistical analysis. Thereby, interanimal variation was avoided and animal numbers could be reduced. Statistical differences were analyzed by Student's *t* test and considered significant at *p *≤ .05 (*), very significant at *p *≤ .01 (**), and highly significant at *p* ≤ .001 (***). All data were presented as mean ± standard error of the mean (SEM), *n* represents the number of mice.

## 3 Results

### 3.1 Preservation of the organotypic environment in cultured hippocampal slices

**Figure 1:**
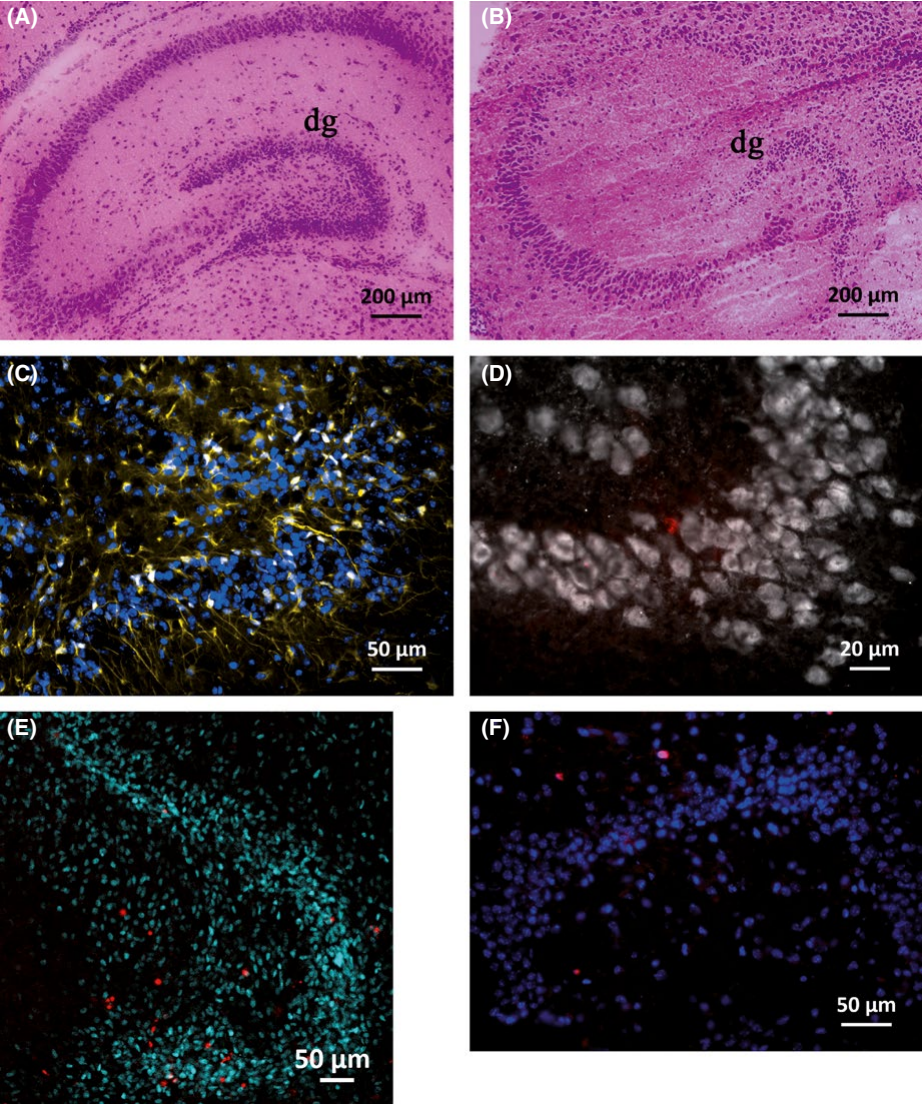
Stainings of cryosectioned brain tissue and hippocampal tissue slices. Hematoxylin–eosin staining of the entorhinal–hippocampal structure in sections from freshly prepared brains (A) and 3 weeks cultured tissue slices (B) of p5 mice. Sectioned tissue slices from p5 mice after 6 weeks of culture (C, D, F) and 4 days after sham irradiation (E): GFAP (yellow)/DAPI (blue) staining of 16 Gy irradiated slices (C), NeuN (white) and BrdU (red) staining of untreated control slices (D), nestin (cyan)/PI (red) staining of untreated controls (E), and Ki‐67 (red)/DAPI (blue) staining of untreated controls (F)

Hematoxylin–eosin staining revealed that the entorhinal–hippocampal formation was well conserved in tissue slices from p5 mice. The histomorphology of cryosectioned brain tissue immediately after sacrifice (Fig.**** 1A) is very similar to one of the section cut from a tissue slice after 3 weeks of culture (Fig. 1B).

### 3.2 Time course analysis of the nestin‐postive neural progenitor cell pool

**Figure 2:**
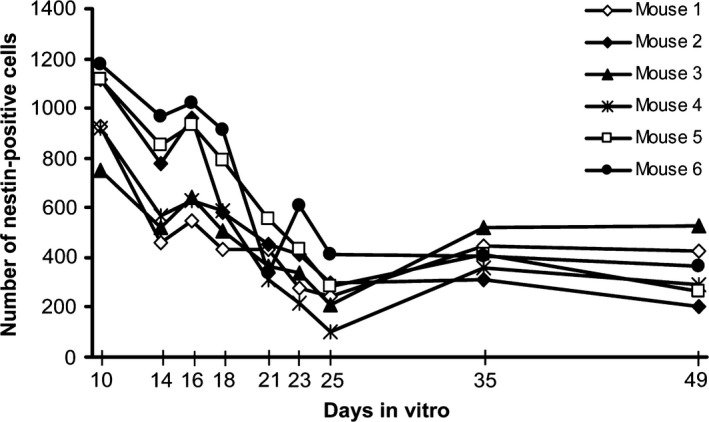
Time‐dependent quantification of nestin‐positive progenitor cells by live imaging microscopy. From six mice (*n* = 6), slices were prepared (day 0) and nestin‐positive cells analyzed at nine different time points for a maximum of 49 days. The number of neural progenitor cells in the dentate gyrus of nonirradiated hippocampal tissue slices declined significantly over time showing a minimum at day 25 (*p* ≤ .001) related to initial (day 10) level

Nestin‐positive progenitor cells were found within the hippocampus mainly in the dentate gyrus, but also in the cornu ammonis regions, vascular zone, and in vascular linings. Expression of nestin was found to be not serum‐dependent; therefore, serum‐based medium was used in all experiments. For time course analysis, quantification of nestin‐positive progenitor cells was performed in the dentate gyrus of nonirradiated hippocampal slice cultures from days 10 to 49 after preparation. Quantification before day 10 was not reasonable, because the wound‐healing processes avoided high‐quality imaging and would have disturbed the results. Live imaging microscopy revealed morephasic shrinkage of the progenitor pool over time. An initial decline of nestin‐positive cells (days 10–14) was interrupted by a short peak at day 16, which was followed by a further drop reaching a minimum at day 25 with a total reduction in neural progenitor cells by 74.1 ± 4.3% (*n *= 6, *p* ≤ .001) related to initial (day 10) level. From there onward, the pool of nestin‐positive cells recovered slightly and remained almost constant reaching about 37 ± 7.8% of the initial (day 10) level at the end of the observation period (Fig.**** 2).

After 2.5 weeks in culture, 45 ± 9.6% of nestin‐positive cells stained also positive for GFAP. This indicates that about half of the nestin‐positive progenitor cell population belongs to the resting putative stem cell pool (type‐1 cell) with no lineage commitment, whereas the other half may belong to type 2a/b (Kempermann, Jessberger, Steiner, & Kronenberg, 2004). In cryosections from freshly prepared brains, 400 nestin‐positive cells in a p5 mouse but only 19 in a p35 mouse were counted (not illustrated).

### 3.3 Effect of irradiation on nestin‐positive neural progenitor cells, live imaging analysis

Before irradiation, the completion of wound healing was confirmed by the measurement of inflammatory cytokines (IL6, KC, MCP‐1) in the slice culture supernatants. Cytokine levels declined after 7 days of slice culture by 92%, 92%, and 58%, respectively, compared to day 1 after slice preparation. Repetitive analysis after 14 days revealed that cytokine release remained at this low level (reduction by 97%, 93%, and 75%, respectively).

**Figure 3:**
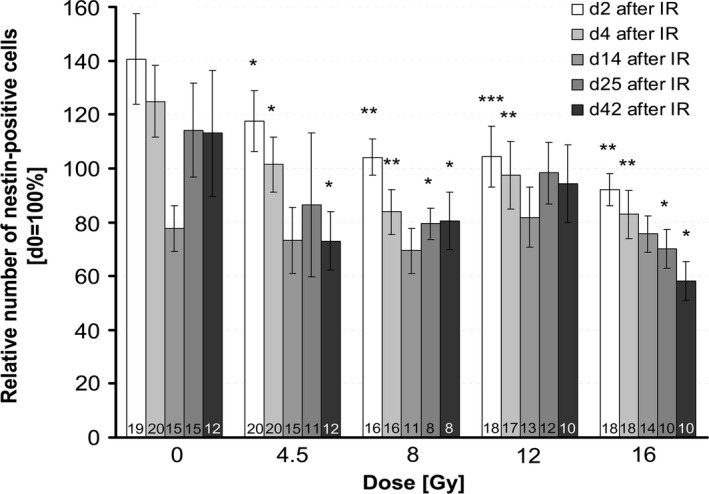
Dose‐dependent quantification of nestin‐positive progenitor cells by live imaging. The number of nestin‐positive cells after irradiation with 4.5, 8, 12, and 16 Gy was analyzed 2, 4, 14, 25, and 42 days after irradiation. Values were normalized to their initial value at day 0 = 100% (before irradiation) to account for slice‐specific (intra‐animal) variation. Significance levels were related to sham‐irradiated controls at the respective day after ionizing radiation to account for the age‐dependent decline of progenitor cells, mean ± SEM,* p *≤ .05 (*), *p *≤ .01 (**), *p *≤ .001 (***). The number of mice (*n*) included in the analysis is presented in each bar

In the applied radiation dose range (4.5, 8, 12, and 16 Gy), the number of nestin‐positive cells within the dentate gyrus was significantly reduced in irradiated slices compared to sham‐irradiated slices during the whole observation period of 42 days (Fig.**** 3, nestin‐staining of untreated control is shown in Fig. 1E). Dose dependency was clearly seen at early time points (days 2 and 4 after ionizing radiation [IR]). The decline of nestin‐positive cells was progressive without recovery already at a relatively low dose of 4.5 Gy within 42 days after irradiation. The most intense decrease was found after application of the highest dose (16 Gy), resulting in 58 ± 7% nestin‐positive cells compared to sham‐irradiated control (100%) 42 days after irradiation. The reduction in progenitor cells at day 25 of culture corresponds roughly to the drop of nestin‐positive cells seen on day 14 after irradiation (0 Gy group, Fig.**** 2). We assume that the lacking effect of irradiation at this time point might be caused by this overlaying event.

### 3.4 Effect of irradiation on neuroglial (GFAP‐positive) cells and mature neurons (NeuN‐positive cells)

**Figure 4:**
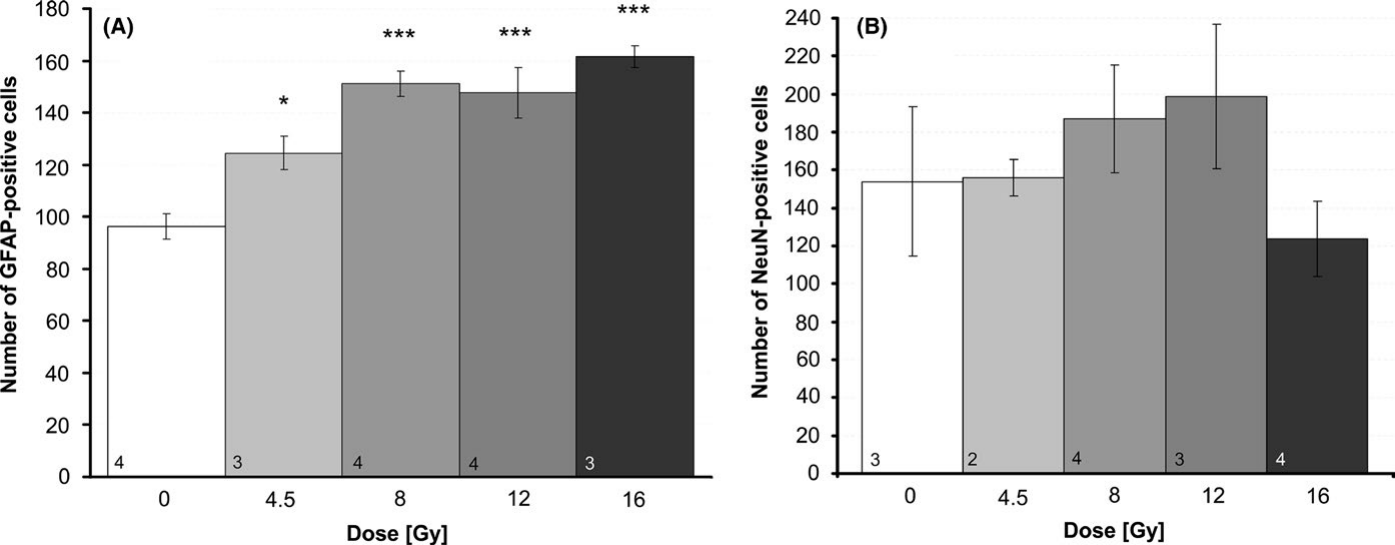
Immunofluorescence analysis of differentiated neuronal cells. (A) Neuroglia cells were identified by GFAP and (B) mature neurons by NeuN surrogate markers in serial cryosections of hippocampal slices 42 days after irradiation. All data are absolute values of stained cells per section, mean ± SEM,* p *≤ .001 (***). The number of mice (*n*) included in the analysis is presented in each bar

On average, there were 96.23 ± 10.19 GFAP‐positive cells within the dentate gyrus of sham‐irradiated controls. GFAP immunoreactivity was slightly enhanced at 4.5 Gy, but significantly increased at 8 Gy (151 ± 9, *p* ≤ .001), 12 Gy (148 ± 13, *p* ≤ .001), and 16 Gy (162 ± 7, *p* ≤ .001), but not at 4.5 Gy 42 days after irradiation (mean ± SEM, *n *≥ 3, Fig. 4A). GFAP staining of untreated control is presented in Fig. 1C.

The number of NeuN‐positive (4.5 Gy: 154 ± 29, 8 Gy: 187 ± 27, 12 Gy: 199 ± 34, 16 Gy: 124 ± 18) mature neurons did not change significantly after irradiation compared to 0 Gy (154 ± 29, mean ± SEM, *n* = 2–4; Fig. 1D staining of untreated control, Fig. 4B graph).

### 3.5 Effect of irradiation on cell survival and proliferation

**Figure 5:**
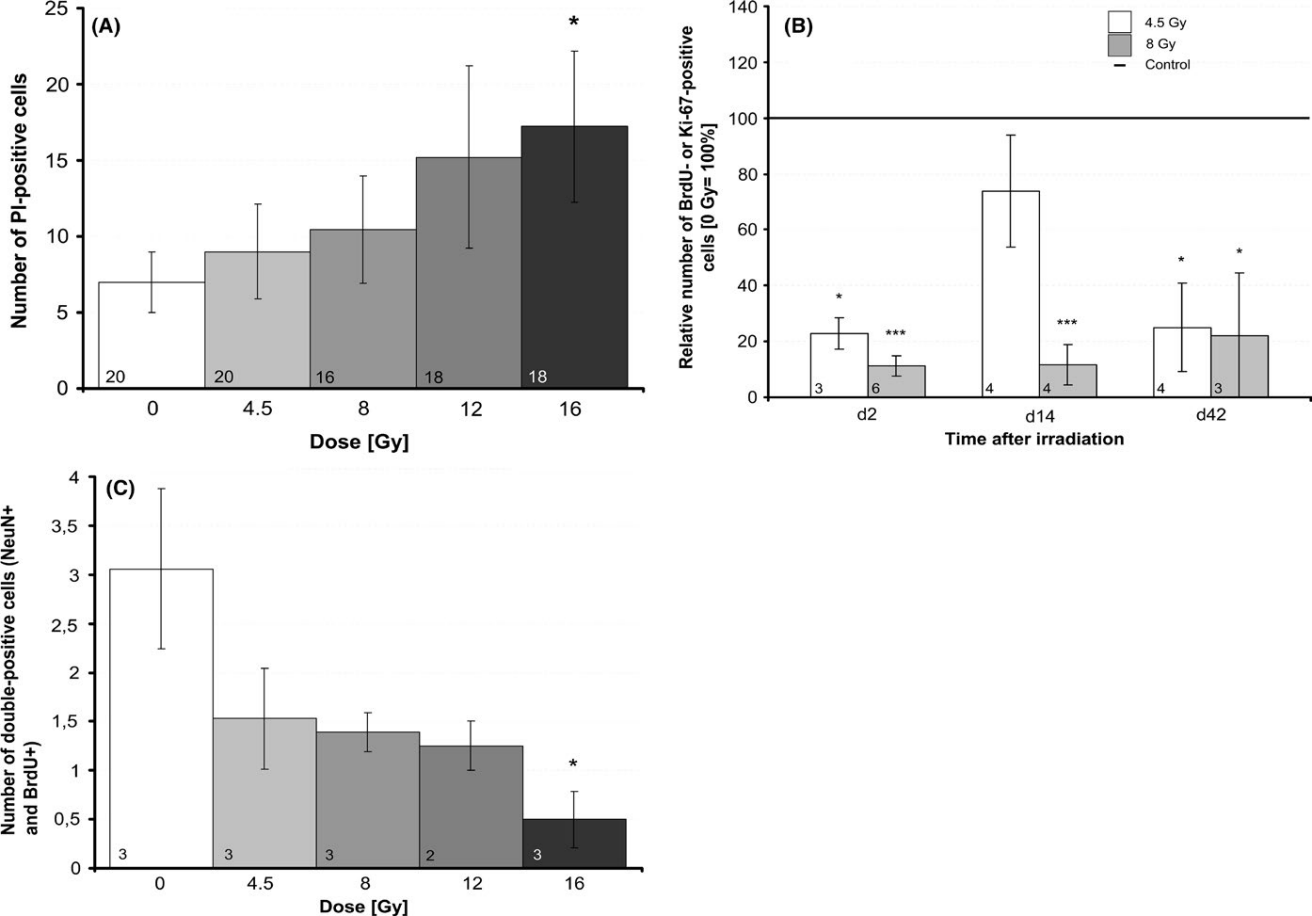
Quantification of cell proliferation and cell death. (A) Cell death was assessed 4 days after irradiation by live imaging analysis of propidium iodide‐positive cells and compared to sham‐irradiated controls, mean ± SEM,* p *≤ .05 (*). The number of mice (*n*) included in the analysis is presented in each bar. (B) Cell proliferation was quantified on days 2, 14, and 42 by uptake of BrdU/Ki‐67. Slices were BrdU pulse labeled on day 2. On day 14 or day 42, slices were fixed, cryosections prepared, and immunofluorescence stained with anti‐BrdU antibody (to analyze cell proliferation on day 2) and with anti‐Ki‐67antibody to analyze the cell proliferation on day 14 or 42 (endpoint). Significance levels were related to sham‐irradiated controls (line = 100%), *n* = 3, mean ± SEM,* p *≤ .05 (*), *p* ≤ .01 (**), *p* ≤ .001 (***). (C) The number of BrdU/NeuN double labeled cells (BrdU‐pulse labeled 2 days after irradiation) was evaluated within the hippocampus 42 days after irradiation to evaluate the development of mature neurons from proliferating progenitors, *n* = 3, mean ± SEM,* p *≤ .05 (*)

To quantify the influence of irradiation on cell survival in the dentate gyrus, we analyzed the number of dead cells after PI uptake by live imaging microscopy, 4 days after irradiation (Fig. 1E, staining of untreated control). The number of PI‐positive cells increased dose dependently after irradiation, reaching significance at a dose of 16 Gy (17 ± 5, mean ± SEM, *n* = 18, *p* ≤ .05) compared to sham‐irradiated control slices (7 ± 2, *n *= 20) (Fig. 5A). To determine the immediate effect of irradiation on cell proliferation, BrdU pulse‐labeled cells were evaluated within the dentate gyrus. Immunofluorescence staining revealed a significant decline of BrdU‐positive cells 0–48 hr after irradiation from 11.67 ± 2.04 (mean ± SEM, *n* = 6) in the sham‐irradiated controls to 2.67 ± 0.67 (*n *= 3, *p* ≤ .01) after 4.5 Gy and to 1.33 ± 0.42 (*n* = 6, *p* ≤ .001) after irradiation with 8.0 Gy. Endpoint proliferation (day 14 and day 42 after irradiation) was estimated by immunofluorescence staining of sectioned slices for Ki‐67, a nuclear‐located marker of proliferation. Ki‐67 labeling was evaluated in dentate gyrus at day 14 and day 42 after IR showing persistently decreased proliferation at both doses (Fig. 5B). Staining of untreated control slices is shown in Fig. 1F.

To investigate how neurogenesis is affected by IR, cells (BrdU pulse labeled at day 2 after IR) were stained in sectioned slices at day 42 after IR for BrdU to detect proliferation, and for NeuN to detect newborn neurons. Double‐labeled cells within the hippocampus were counted in sections of three mice (*n* = 3). An IR dose‐dependent reduction of NeuN/BrdU double‐labeled cells was detected and became significant at 16 Gy, *p* ≤ .01 (Fig. 5C graph). Fig. 1D shows NeuN and BrdU staining of an untreated control slice.

### 3.6 Effect of resveratrol on irradiated neural progenitor cells

**Figure 6:**
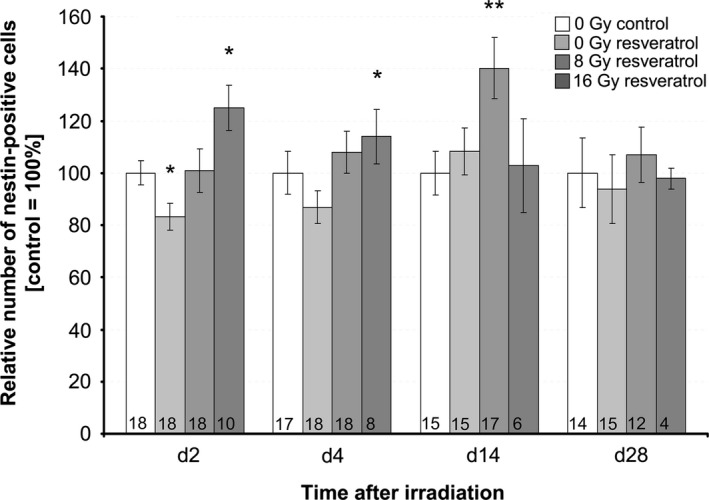
Effect of resveratrol, 15 μmol/L, and irradiation on nestin‐positive neural progenitors. The number of nestin‐positive cells was analyzed at days 2, 4, 14, and 28 after irradiation with 8 or 16 Gy in resveratrol‐treated versus untreated, irradiated‐only slices (white bar = 100%), mean ± SEM,* p *≤ .05 (*), *p *≤ .01 (**). The number of mice (*n*) included in the analysis is presented in each bar. Values were normalized to their initial value (d 0) to account for slice‐specific (intra‐animal) variation and to irradiated‐only control slices at the respective day

To assess the neuroprotective potential of resveratrol on neural progenitor cells, slices were incubated with and without 15 μmol/L resveratrol during irradiation and nestin‐positive cells were counted within the dentate gyrus at four different time points afterward (Fig. 6). In sham‐irradiated slices, treatment with resveratrol compared to untreated control transiently decreased the nestin‐positive cells by 17%, *p* ≤ .05, 2 days after sham irradiation.

In irradiated slices, a significantly larger pool of nestin‐positive neural progenitor cells was maintained in slices with versus without resveratrol. After irradiation with 8 Gy, more nestin‐positive cells were counted in slices treated with resveratrol than without over the whole observation period (28 days). This effect became significant at day 14 after irradiation, when nestin‐positive cells reached 140% ± 12% (*n *= 17, *p* ≤ .01) of controls (100% ± 8%, *n* = 13). Also, after irradiation with 16 Gy, the number of nestin‐positive cells was significantly higher in resveratrol‐treated slices (day 2: 125% ± 9%, *n *= 10, *p *≤ .05, day 4: 114% ± 10%, *n *= 8, *p* ≤ .05) than in untreated control slices. However, at this high dose, the radio/neuroprotective effect was not maintained at later time points (days 14 and 28).

## 4 Discussion

Treatment‐related neurocognitive impairment is a major clinical problem in neuro‐oncology.

An important cause is the radiation‐induced decline of hippocampal neurogenesis. In contrast to mature neurons, which are relatively radioresistant, neural precursor cells are particularly vulnerable to IR. Therefore, clinicians question if a reduction in the radiation exposure of the hippocampus to total doses below 10 Gy, which can be achieved by modern sparing techniques and which is currently evaluated in several clinical studies, may suffice to prevent serious adverse events. Innovative drugs providing the capacity to increase the resistance of critical cell populations against irradiation and at the same time not compromising the antitumoral effect of radiation therapy would be of great benefit.

In this study, we used entorhinal–hippocampal tissue slice cultures as an organotypic ex vivo model which can be kept for a long period to assess radiation‐induced and neuroprotective effects on neurogenesis. They maintain their morphological (Fig. 1) and functional (Kluge et al., 1998; Raineteau, Rietschin, Gradwohl, Guillemot, & Gahwiler, 2004) integrity over several weeks. By time course analysis over 49 days, we revealed a decline of nestin‐positive neural progenitor cells in the dentate gyrus. Although, to our knowledge, its morephasic dynamic has not yet been described so far, this finding supports observations that these organotypic cultures age in vitro similarly to mice in vivo (Fukuda et al., 2005). In fact, our nestin‐positive cell counts of p5 and p35 mice revealed a strong age‐dependent reduction of the nestin‐positive stem cell pool in vivo. The distribution of nestin‐expressing progenitor cells in our slice cultures correlates with results from Raineteau et al. (2004), who used neurogenin‐2 as a marker to identify neural stem cells. Nestin immunofluorescence was additionally visible in vascular linings as published by others (Mokry et al., 2008). In contrast to neurogenin‐2, nestin expression was not significantly changed by serum‐based versus serum‐free medium, indicating a differential activation profile for these neural stem cell markers.

We chose to irradiate p3–6 mice after circa 14 days in culture, to generate a model which corresponds to children less than 10 years old (Flurkey & Currer, 2004). This age group is characterized by a particularly high vulnerability regarding radiation‐induced cognitive late effects (Merchant, 2009; Palmer et al., 2013) and possibly changes in neurogenesis compared to older children.

From the multiple transgenic mouse lines available for a study of neurogenesis (Dhaliwal & Lagace, 2011), we chose specifically the nestin‐CFPnuc mouse to visualize the effect of IR on quiescent uncommitted putative stem cells as well as on amplifying neural progenitor cells (Encinas et al., 2006; Kempermann et al., 2004; Lendahl et al., 1990).

The first main result of this study was to show that the nestin‐positive neural progenitor cell population in the dentate gyrus is long‐term, possibly even irreversibly damaged by single doses of 4.5, 8, 12, or 16 Gy, going along with decreased cell proliferation and enhanced cell death. No recovery of the neural stem cell pool was found at any dose within 6 weeks. Double staining for BrdU/NeuN confirmed an IR‐induced decline of neurogenesis showing an impaired development of new neuronal cells (NeuN) from proliferating progenitors (BrdU). This is consistent with previous findings showing irradiation‐induced neural precursor cell dysfunction and long‐term decreases in neurogenesis by NeuN/BrdU double labeling (Madsen, Kristjansen, Bolwig, & Wortwein, 2003; Mizumatsu et al., 2003; Monje, Mizumatsu, Fike, & Palmer, 2002).

Most importantly, already a moderate dose of 4.5 Gy caused long‐lasting decreases in nestin‐positive neural progenitor cells, the prerequisite of hippocampal neurogenesis. This dose corresponds to about 10 Gy in a typical fractionated setting (15 fractions, α/β: 3), thus reflecting the dose which is technically feasible in “hippocampus sparing” settings (Gondi et al., 2010). Assuming an α/β ratio of 10 for the proliferating neuronal precursor cell population, an even lower equivalent dose of about 6 Gy would result, at which we may expect to induce damage to neurogenesis. This underlines the need for additional neuroprotective measures beside the technical approaches.

Our results are consistent with findings of Bostrom, Kalm, Karlsson, Hellstrom Erkenstam, and Blomgren (2013) who found an increasing depletion of neurogenesis at 1 week, 7 weeks, and 1 year after whole brain irradiation of postnatal day 14 mice with a single dose of 8 Gy. The stronger decrease in neurogenesis found by this group is surely the result of counting DCX‐positive cells, which mainly reflect the proliferating and therefore more radiosensitive, neural precursor cells. Similarly, Hellstrom et al. (2009) showed that a single whole brain dose of 6 Gy at postnatal day 9 reduced proliferation and neurogenesis in the adult brain (9 weeks after irradiation) of rats, counting nestin/GFAP‐positive neural stem cells (Hellstrom et al., 2009). In young adult mice (8 weeks), Kim et al. (2008) reported that the depletion of neurogenesis (DCX‐positive cells) was reversible 2 weeks after 2 but not 4 Gy whole body irradiation. Within 1 month, however, doses of 4 Gy applied to adult mice (3 months) seem to have reversible effects on neurogenesis (DCX‐positive cells) (Ben Abdallah, Slomianka, & Lipp, 2007) pointing toward differences in the (time to) recovery of juvenile and adult neural progenitor cell pool. In our model, there was no recovery detected. The number of proliferating cells within the dentate gyrus showed an acute and persistent decline already at a dose of 4.5 Gy going along with findings by Rola et al. (2004) in 21‐day‐old mice after 5 Gy irradiation. In parallel, dead cell counts increased dose‐dependently 96 hr after irradiation. Absolute dead cell numbers, however, were relatively low and cannot account on its own for the decline of nestin‐positive cells after irradiation. At higher doses, mitotic catastrophes occurring over the course of multiple cell divisions, however, might lead to delayed cell death. We hypothesize that the long‐term decline of proliferation and nestin‐positive cells might be due to an irradiation‐induced switch of cell division from asymmetric monodifferentiative toward symmetric proliferative (terminal) division (Huttner & Kosodo, 2005). Furthermore, an aberrant differentiation of neural stem cells, changing the ratio of astrocytes and neuronal cells, might enhance the adverse effects of irradiation. Both effects have been described by Monje et al. (2002), in an adult rat model of 10 Gy single‐dose whole brain irradiation with subsequent in vitro cultivation of precursor cells from the hippocampus. After being irradiated, neural stem cells can be triggered toward GFAP‐positive astrocyte differentiation by upregulation of NF‐κB (Ozeki, Suzuki, Suzuki, Ozawa, & Yamashita, 2012) and induction of STAT3 (Bonni et al., 1997), or to reactive gliosis by microglia activation (Hwang et al., 2006). This goes along with our findings of a significant dose‐dependent increase in GFAP‐positive cells within the dentate gyrus. In contrast, the number of mature neurons (NeuN‐positive cells) did not change significantly after irradiation. Although we cannot exclude that NeuN‐positive cells are also reduced by irradiation but recover within 6 weeks, others confirm a relatively low sensitivity of mature neurons toward irradiation (Wu et al., 2010).

The second main result of this study was the finding of a neuroprotective effect of resveratrol on irradiated neural progenitor cells in hippocampal slice cultures.

Keeping in mind the differential effects of resveratrol on tumor versus normal cells and its low toxicity (Fukui et al., 2010; Li et al., 2014; Zhang et al., 2006), resveratrol is an interesting candidate in antitumor therapy. Associated (protective or unwanted) effects of resveratrol on normal neural stem cells, investigated here, are of high relevance for any potential application in brain tumor therapy. Protective actions of resveratrol have been reported on irradiated (4 Gy) hippocampal neurons (Li et al., 2014) in rats, and in irradiated mice by ameliorating hematopoietic stem cell injury (Zhang et al., 2013). In vitro experiments demonstrated that resveratrol may enhance the survival of rat neuronal cells exposed to amyloid‐beta toxicity or nitric oxide (Bastianetto et al., 2015).

In this study, we could show for the first time a neuroprotective effect of resveratrol (15 μmol/L) on 8 Gy irradiated neural progenitor cells during the whole observation period. The combination of resveratrol and 16 Gy irradiation also significantly reduced the irradiation‐induced decrease in nestin‐positive neural progenitor cells 2 and 4 days after irradiation, indicating protection against acute cell death. However, on days 14 and 28 after irradiation at 16 Gy, resveratrol showed no neuroprotective effects anymore. Possibly, late events in the resting stem cell population cannot be prevented by resveratrol after such high irradiation doses. In contrast, resveratrol (15 μmol/L) led to a slight decline of neural stem cells within the dentate gyrus in sham‐irradiated control hippocampal slices. This goes along with findings that resveratrol at a concentration of 10–50 μmol/L leads to a reduction of proliferating neural precursor cells in young mice. Lower concentrations of resveratrol (1 μmol/L) did not show adverse effects (Park, Kong, Yu, Mattson, & Lee, 2012). These data indicate that high resveratrol concentrations should be restricted to irradiated patients only.

We have already shown that resveratrol decreases the survival in the most common human medulloblastoma cell lines synergistically with irradiation (manuscript in preparation) and on its own (Patties, Kortmann, & Glasow, 2013). The resveratrol concentration of 15 μmol/L used in the present study led to a 30% reduction of metabolic activity in medulloblastoma cell lines (Patties et al., 2013). It should be feasible for a treatment in humans as it is still well below the concentration of 40 μmol/L, which is maximally achievable after intravenous injection (Asensi et al., 2002). In a fractionated setting, when repetitive doses are given, resveratrol concentrations can be reduced to 2 μmol/L (own observations) which can be reached by oral administration in humans (Boocock et al., 2007).

The knowledge about the molecular targets of resveratrol might partially explain its differential action on irradiated/nonirradiated normal and tumor cells, whereby its effects strongly depend on cell type, animal age, dose, and study duration.

We assume that the neuroprotective action of resveratrol on irradiated neural progenitor cells described here might be a combinatorial result: first, of its direct and its cellular ROS scavenging ability (Leonard et al., 2003; Mokni, Elkahoui, Limam, Amri, & Aouani, 2007; Bastianetto et al. 2015); second, of its anti‐inflammatory activity, for example, via suppression of (nuclear factor kappa B) NF‐κB signaling (Li et al., 2014). Besides, additional genes and processes might be modulated, for example, leading to accelerated recovery from IR‐induced DNA strand breakage (Denissova, Nasello, Yeung, Tischfield, & Brenneman, 2012).

The situation seems to be different in normal cells, not exposed to irradiation. Here, resveratrol‐induced SIRT1 activation may suppress self‐renewal of neural hippocampal stem cells (Ma et al., 2014). Moreover, resveratrol activates 5′‐adenosine monophosphate‐activated protein kinase (AMPK) which inhibits normal neural stem and progenitor cell growth through suppression of CREB (cAMP response element‐binding protein) and BDNF signaling (Park et al., 2012).

The anticancer activities of resveratrol include the inhibition of proliferation and tumorigenicity as well as the induction of apoptosis in tumor cells. These processes involve, for example, the downregulation of PI3K/Akt/NF‐κB signaling pathway and the activation of AMPK and of SIRT2 (Lu et al., 2009; Rattan, Giri, Singh, & Singh, 2005; Sayd et al., 2014). Furthermore, resveratrol has been shown to sensitize brain tumor cells to IR (Lu et al., 2009), possibly by its pro‐oxidant Cu(II)‐dependent effects. As cancer cells have been shown to contain elevated levels of copper, they might be more sensitive to the proapoptotic and cell‐damaging effects of resveratrol than normal cells (Gagliano et al., 2010).

## 5 Conclusion

Irradiation doses from 4.5 to 16 Gy reduced the neural progenitor pool irreversibly, possibly by a combinatorial effect of proliferation inhibition, cell death induction, and aberrant differentiation. As dose and age of the ex vivo hippocampal slice culture model correspond to a “hippocampal sparing” therapeutic irradiation in infantile patients, these results underline that additional measures are necessary to prevent radiation‐induced adverse effects on neurogenesis in tumor patients. Resveratrol was able to reduce the irradiation‐induced decline of progenitor cells warranting further investigations to verify its neuroprotective potential in vivo.

## Funding Information

The project was funded by the Deutsche Forschungsgemeinschaft grant GL 780/2‐1, 610774.

## Conflict of Interest

None declared.
